# Evolution of Esophageal Cancer Incidence Patterns in Hong Kong, 1992-2021: An Age-Period-Cohort and Decomposition Analysis

**DOI:** 10.3389/ijph.2024.1607315

**Published:** 2024-08-07

**Authors:** Lijun Wang, Jianqiang Du, Haifeng Sun

**Affiliations:** ^1^ Department of Orthopaedics, Xijing Hospital, Fourth Military Medical University, Xi’an, China; ^2^ Key Laboratory of Biomedical Information Engineering, Ministry of Education, School of Life Science and Technology, Xi’an Jiaotong University, Xi’an, China; ^3^ Third Department of Medical Oncology, Shaanxi Provincial Cancer Hospital Affiliated to Medical College of Xi’an Jiaotong University, Xi’an, China

**Keywords:** esophageal cancer, incidence, period analysis, cohort effect, demographic factors

## Abstract

**Objective:**

To elucidate the historical trends, underlying causes and future projections of esophageal cancer incidence in Hong Kong.

**Methods:**

Utilizing the Age-Period-Cohort (APC) model, we analyzed data from the Hong Kong Cancer Registry (1992–2021) and United Nations World Population Prospects 2022 Revision. Age-standardized incidence rates were computed, and APC models evaluated age, period, and cohort effects. Bayesian APC modeling, coupled with decomposition analysis, projected future trends and identified factors influencing incidence.

**Results:**

Between 1992 and 2021, both crude and age-standardized incidence rates of esophageal cancer witnessed significant declines. Net drifts exhibited pronounced downward trends for both sexes, with local drift diminishing across all age groups. Period and cohort rate ratios displayed a consistent monotonic decline for both sexes. Projections indicate a continued decline in esophageal cancer incidence. Population decomposition analysis revealed that epidemiological changes offset the increase in esophageal cancer cases due to population growth and aging.

**Conclusion:**

The declining trend of esophageal cancer in Hong Kong is influenced by a combination of age, period, and cohort. Sustaining and enhancing these positive trends requires continuous efforts in public health interventions.

## Introduction

Esophageal cancer ranks as the seventh most frequently diagnosed cancer and the sixth leading cause of cancer-related mortality, accounting for an estimated 604,000 new cases and 544,000 deaths in 2020, constituting 3.1% of all cancer cases and 5.5% of cancer-related deaths globally [1]. East Asia bears the highest incidence of esophageal cancer [2, 3], where, in China, it stands as the fifth most prevalent cancer and the fourth leading cause of cancer-related deaths in 2020 [4]. Notably, Hong Kong, as a special administrative region of China, reports a relatively low incidence, ranking 20th in cancer incidence in 2020 [5]. Over the last three decades, there has been a significant reduction in the incidence of esophageal cancer in Asia, including Hong Kong [1, 5–7].

Esophageal cancer is a heterogeneous disease that can be classified into two main histological subtypes, squamous cell carcinoma (SCC) and adenocarcinoma (AC), with distinct etiologies [1, 8, 9]. The geographical distribution of these subtypes varies significantly. SCC is the predominant subtype in China, representing over 85% of cases [10], primarily attributed to tobacco smoking and alcohol consumption [2, 3]. Conversely, AC constitutes approximately two-thirds of cases in Western high-income countries and is linked to factors such as being overweight, gastroesophageal reflux disease, and Barrett’s esophagus [1, 6].

Various factors, including demographic changes and risk factor exposure, contribute to esophageal cancer incidence [1, 3]. In recent years, changing social and cultural norms have led to decreased smoking and alcohol consumption rates, potentially contributing to a decline in esophageal cancer incidence in East Asian countries [6, 11–13]. However, population aging, a significant demographic change in many East Asian countries, including Hong Kong, has increased the proportion of individuals in age groups with a higher risk of developing esophageal cancer. Therefore, the burden and trends of esophageal cancer result from a complex interplay between epidemiological and demographic factors. Previous literature has documented a notable decline in SCC incidence in East Asia, including China and Japan, from the 1980s to the 2010s [13, 14]. This decline is likely attributed to reduced smoking rates, improved dietary habits, and better control of risk factors such as alcohol consumption [14, 15]. These positive trends highlight the impact of public health interventions and lifestyle changes in reducing esophageal cancer incidence in the region. Understanding the reasons behind these trends is crucial for developing effective prevention and early detection strategies to further reduce the burden of esophageal cancer on the population.

The primary objective of this study was to analyze patterns and trends in esophageal cancer incidence in Hong Kong using the age-period-cohort (APC) model. This model enables the identification of the impact of birth cohort and diagnosis period on long-term trends in esophageal cancer incidence. In addition to analyzing historical data, we projected the trends in esophageal cancer incidence in Hong Kong up to 2030. Finally, we aimed to quantitatively decompose the contribution of demographic and epidemiological factors to the increase in new cases of esophageal cancer. Our findings offer valuable insights into the underlying causes of esophageal cancer incidence trends in Hong Kong and may inform the development of effective prevention and early detection strategies to mitigate the impact of esophageal cancer on the population.

## Methods

### Study Population and Population Data

The study focused on individuals newly diagnosed with esophageal cancer in Hong Kong between 1992 and 2021. Data, including the year of diagnosis, age at diagnosis, and gender, were sourced from the Hong Kong Cancer Registry (HKCaR) [16], a population-based cancer registry operational since 1963. The HKCaR maintains the highest data completeness standard among developed countries, as recognized by the International Agency for Research on Cancer (IARC). Esophageal cancer cases were identified using the International Classification of Diseases 9th Revision code 150 and 10th Revision code C15. Patients under 20 were excluded to concentrate on the primary age groups at risk.

Population estimates and projections from the United Nations (UN) World Population Prospects 2022 Revision [17] were employed to provide reliable demographic data. This official source ensured universally recognized estimates of population size and demographic trends. Age-standardized incidence rates, using the direct method with the World Health Organization (WHO) standard population from 2000 [18], facilitated accurate comparisons of incidence rates within Hong Kong and with other regions or countries using the same standard population.

### Statistical Analysis

Age-Period-Cohort (APC) models [19] were utilized to assess the influence of age, period, and birth cohort on esophageal cancer incidence trends. APC models are commonly used to analyze cancer incidence trends over time and allow for the estimation of age, period, and cohort effects on cancer incidence. The count of cancer cases and population data were collected and tabulated into fourteen 5-year age groups (ranging from 20–24 years to 85+ years) and six 5-year calendar periods (1992–1996 to 2017–2021) to ensure consistency across age and time-period groups. Nineteen birth cohorts based on age and period groups (1907–1997) were defined. APC models estimated net drift (representing the annual percentage change in the expected age-adjusted rate over time) and local drift (annual percentage change over time for a specific age group). Incidence rate ratios (RRs) with 95% confidence intervals were used to present birth cohort and period effects, with the first age and period range set as the reference points [20–23]. The National Cancer Institute’s Age-Period-Cohort web tool facilitated model fitting, and Wald tests assessed variable significance [20]. Separate analyses were conducted for each sex, with all tests being two-sided and a significance level of 0.05.

To enhance the reliability of future trend projections (2022–2030), the study employed the Bayesian APC framework [24]. This framework incorporated prior knowledge, historical trends, and expert opinions to estimate uncertainty in projections. The integrated nested Laplace approximation (INLA) served as a computational tool, offering a faster and more accurate alternative to Markov Chain Monte Carlo methods, ideal for large-scale projects like population projections. The BAPC package in R was used to fit the Bayesian APC model and perform the projections [24]. This package provides the estimation of posterior distributions of model parameters and reliable uncertainty estimates for the projections. Using this framework and computational tool, the study aimed to provide a more robust projection of future trends in esophageal cancer incidence and new cases in Hong Kong.

A decomposition analysis from 1992 to 2030 explored factors influencing changes in esophageal cancer incidence in Hong Kong. Three main factors were considered: population growth, population ageing, and age-specific incidence rates (epidemiological changes) [25]. Epidemiological changes, driven by alterations in risk factors and healthcare, were isolated from the effects of population growth or ageing. A validated algorithm, insensitive to the choice of reference year or decomposition order, was employed for this analysis [26, 27]. Further details on decomposition and projection are provided in Supplementary Appendix S1, S2. Data processing and analysis were executed using the R programming language, version 4.2.3.

## Results

### Trends in the Incidence of Esophageal Cancer, 1992 to 2021

Between 1992 and 2021, a total of 13,832 esophageal cancer cases were recorded in Hong Kong, with 80.3% in men and 19.7% in women. The age-standardized incidence rates significantly declined from 1992 to 2021 for both men (14.8–4.4 per 100,000) and women (3.3–0.8 per 100,000). Crude incidence rates also decreased over this period.

During this period, there was a notable reduction in the number of esophageal cancer cases in both men and women in Hong Kong. Specifically, the number of cases in men decreased from 430 in 1992 to 325 in 2021. Similarly, for women, the number of cases dropped from 109 in 1992 to 72 in 2021, indicating a notable decrease in the incidence of esophageal cancer in the female population.

### APC Modeling in the Incidence of Esophageal Cancer, 1992 to 2021

**FIGURE 1:**
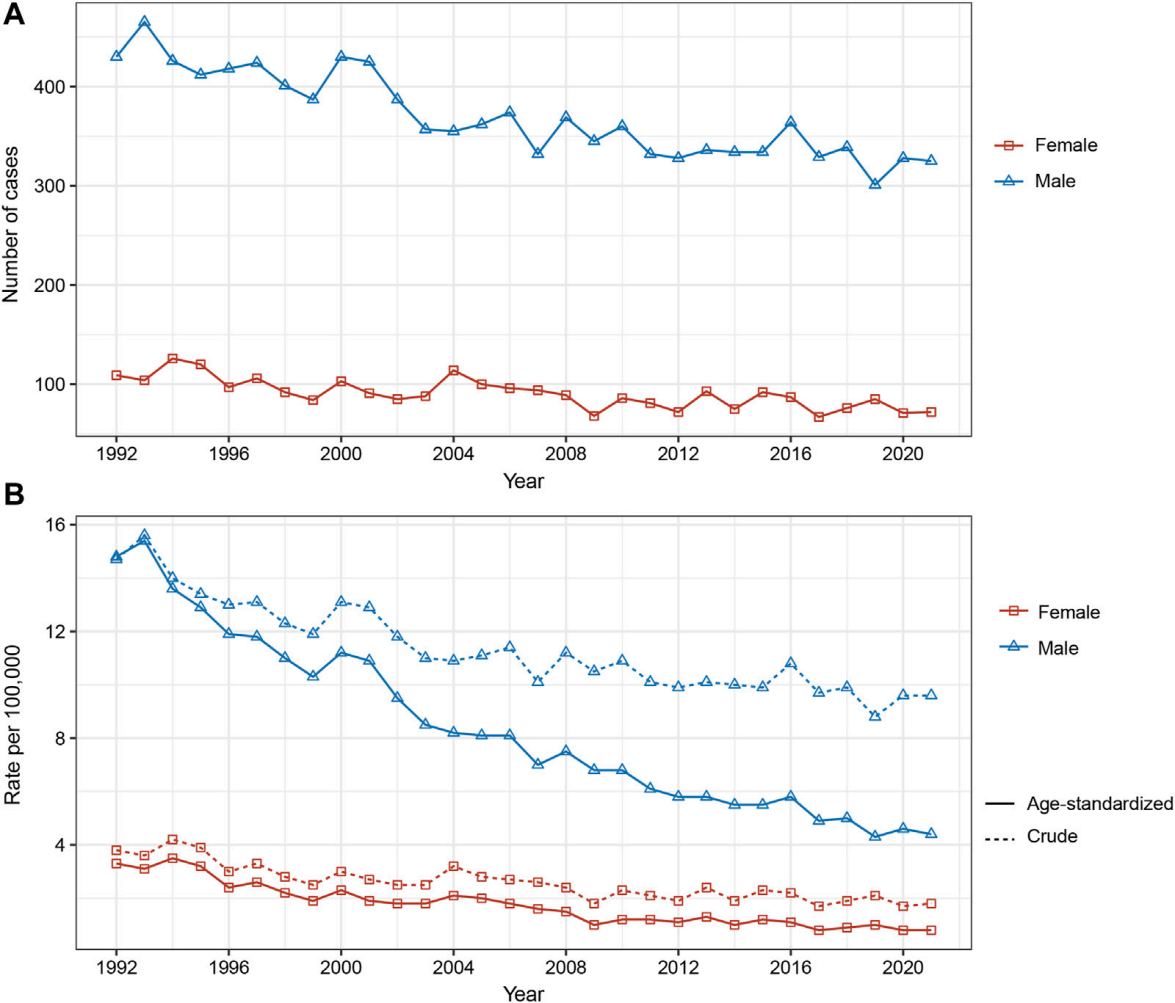
Changes in incidence rate and new cases of esophageal cancer in Hong Kong, 1992–2021. **(A)** New esophageal cancer cases. **(B)** Age-standardized and crude incidence rate (Hong Kong, China. 2024).

**FIGURE 2:**
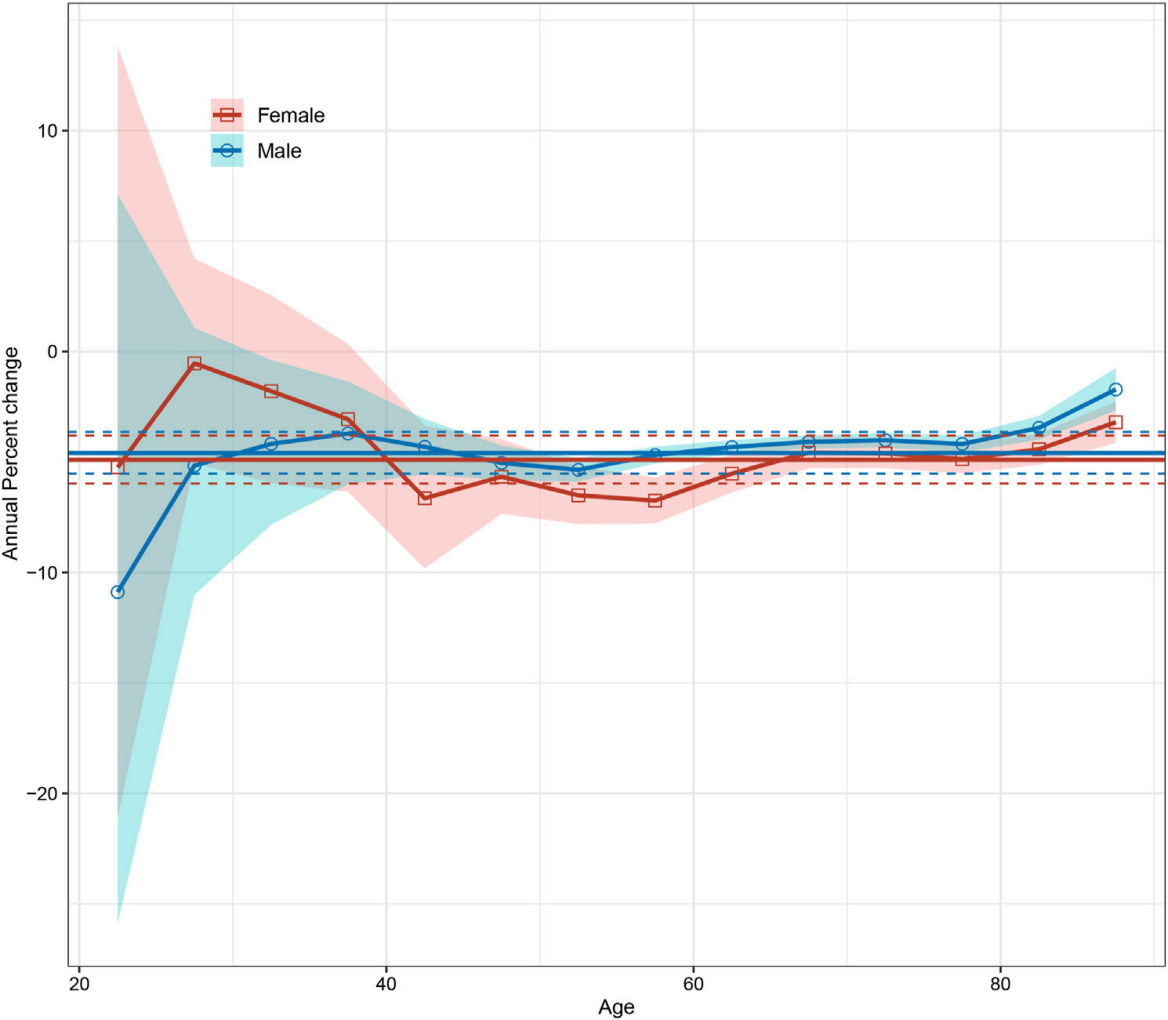
Local drifts with net drift values for esophageal cancer incidence in Hong Kong from 1992 to 2021. The horizontal solid line and corresponding dashed lines represent the net drift and its 95% confidence intervals, while the curve and corresponding shaded area represent the local drift and its corresponding 95% confidence intervals (Hong Kong, China. 2024).

APC modeling revealed a significant downward trend in net drifts of esophageal cancer incidence for both men and women, with a decrease of −4.59% (95% confidence interval [CI]: −5.53% to −3.64%) per year for men and −4.89% (95% CI: −5.98% to −3.80%) per year for women (Figure 1). The local drift, which considers changes in age-specific incidence rates, decreased across all age groups, with a more substantial decline in those aged 40 to 60. Incidence rates were lower in younger age groups, peaked around 60–70, and declined. However, there was some uncertainty in the estimates for the younger age groups, as indicated by the wide 95% confidence intervals (Figure 2).

**FIGURE 3:**
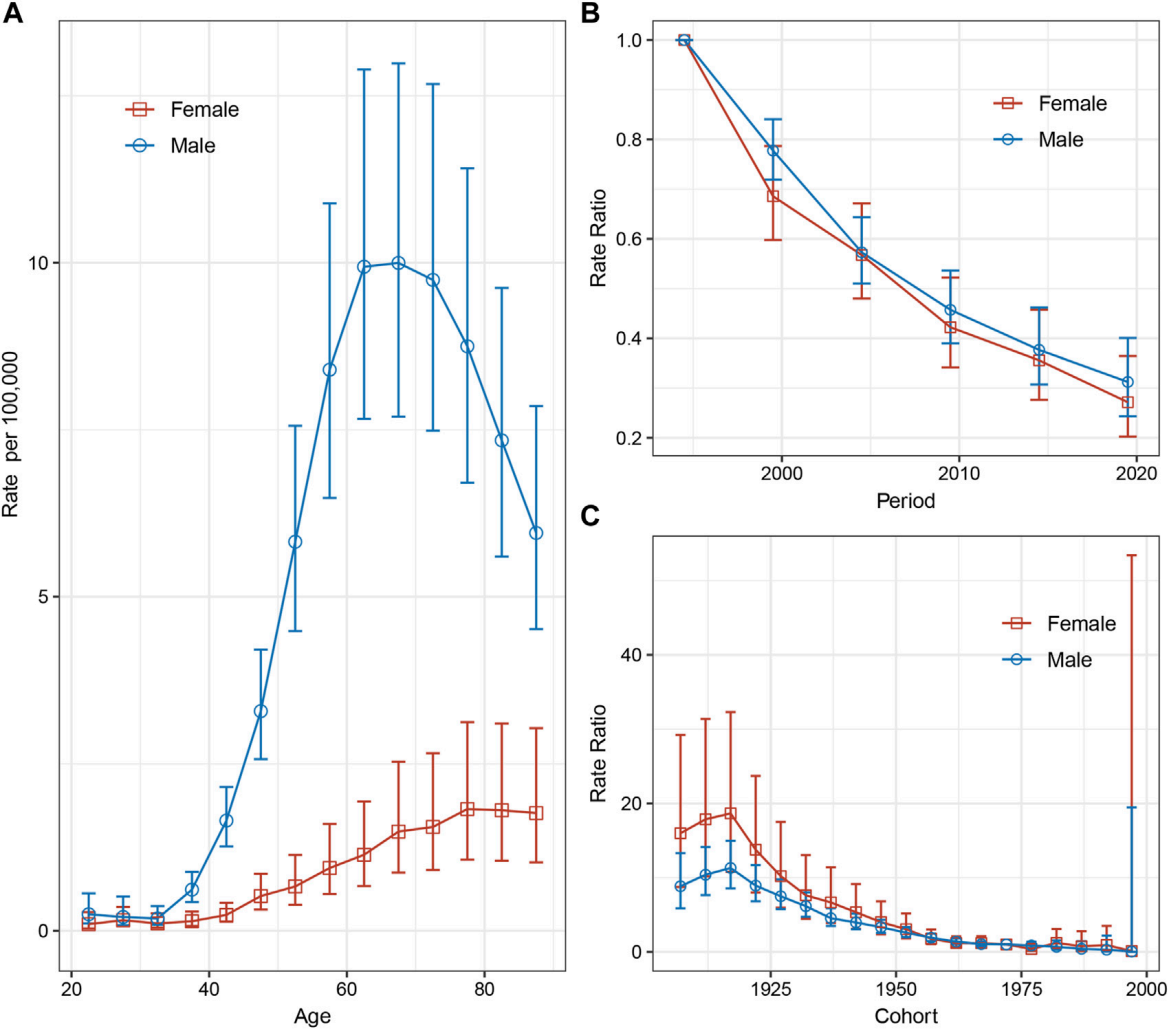
Age, calendar period, and birth cohort effects with the corresponding 95% confidence intervals on esophageal cancer incidence rates by sex, Hong Kong, 1992–2021. **(A)** Longitudinal curves of fitted age-specific rates in reference cohort adjusted for period effects. **(B)** Rate ratios in each period relative to the reference period, adjusted for age and non-linear cohort effects. **(C)** Rate ratios in each cohort relative to reference cohort, adjusted for age and non-linear period effects (Hong Kong, China. 2024).

Upon adjusting for period deviations, the analysis revealed distinct patterns in the incidence of esophageal cancer in relation to age and gender. The incidence of esophageal cancer was observed to be lower in younger age groups, particularly those under 35 years old. As age increased, there was a subsequent rise in the incidence, reaching a peak around the age range of 60–70 years. Following this peak, there was a decline in the incidence of esophageal cancer. Additionally, the data indicated a gender difference in the incidence rates, with men exhibiting a significantly higher incidence rate of esophageal cancer compared to women (Figure 3A).

The period rate ratios exhibited a consistent, monotonic decline for both sexes, signaling a notable period effect. This indicates that, over the analyzed period (1992–2021), there was a systematic and downward shift in the incidence rates of esophageal cancer for both men and women (Figure 3B). The cohort rate ratios, however, displayed an initial increase before following a generally decreasing trend for both sexes. This initial increase suggests some variability in the early birth cohorts, but the overall long-term trend indicates a decrease in esophageal cancer incidence (Figure 3C).

The statistical significance of these trends and effects was confirmed by the Wald test, emphasizing the robustness of the observed patterns in both period and cohort effects for both men and women, as detailed in Supplementary Table S1.

### Projection Up to 2030

**FIGURE 4:**
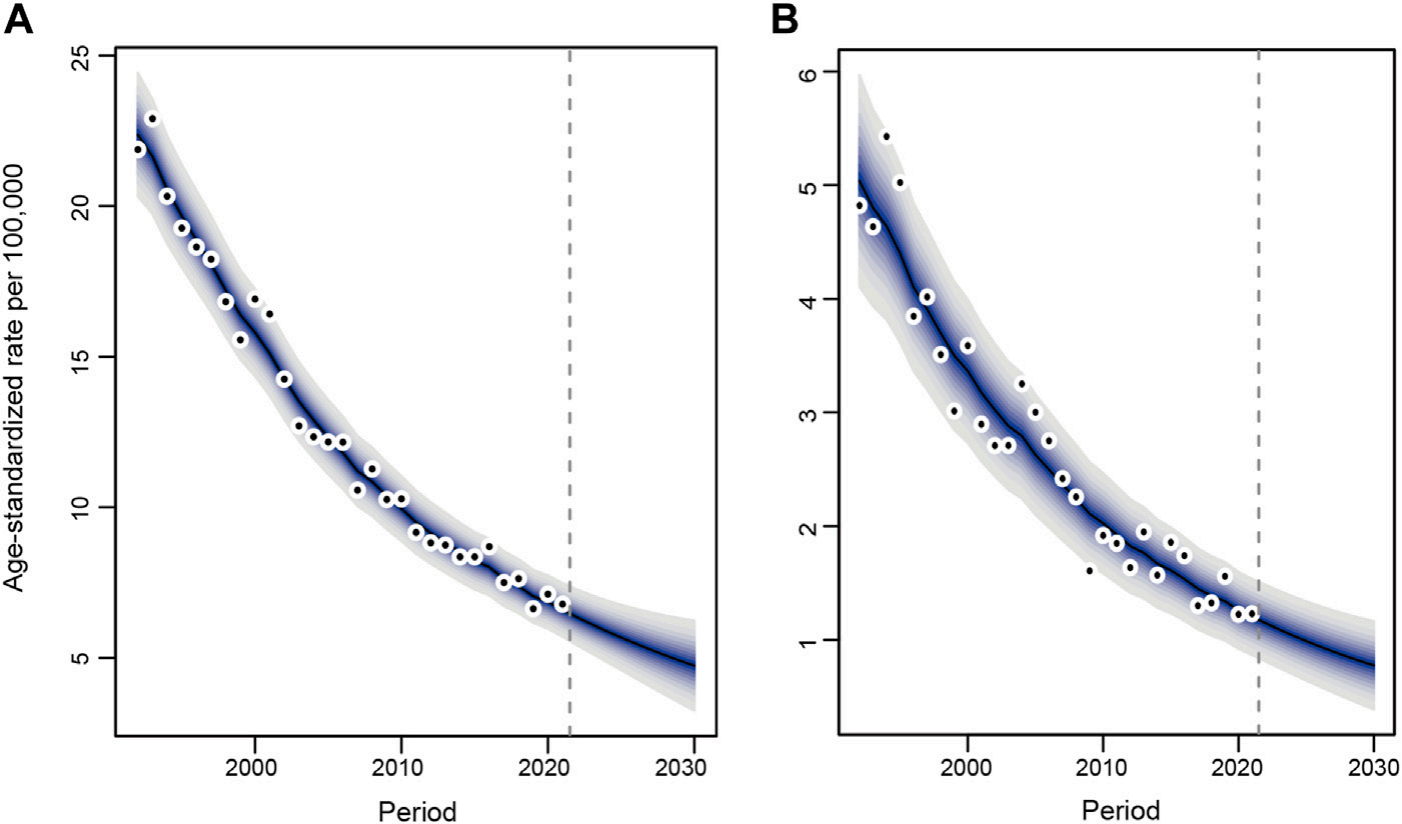
Trends and projected incidence rates for esophageal cancer in Hong Kong. **(A)** Men. **(B)** Women. Dots represent fitted points. Data in the right of the dashed line were projected data. Each lighter shade of blue represents an additional 10% confidence interval (Hong Kong, China. 2024).

The projections up to 2030 indicate a further decrease in new esophageal cancer cases in Hong Kong. Specifically, the expected changes in new cases from 2022 to 2030 are projected to be a decrease from 315 to 277 for men and a decrease from 73 to 64 for women, as detailed in Supplementary Tables S2, S3. Additionally, age-standardized incidence rates are also projected to decline for both sexes, as illustrated in Figure 4. This decline suggests a continued overall reduction in the incidence of esophageal cancer, further supporting the trend observed in the historical data from 1992 to 2021.

### Decomposition of Changes in Esophageal Cancer Cases, 1992 to 2030

**FIGURE 5:**
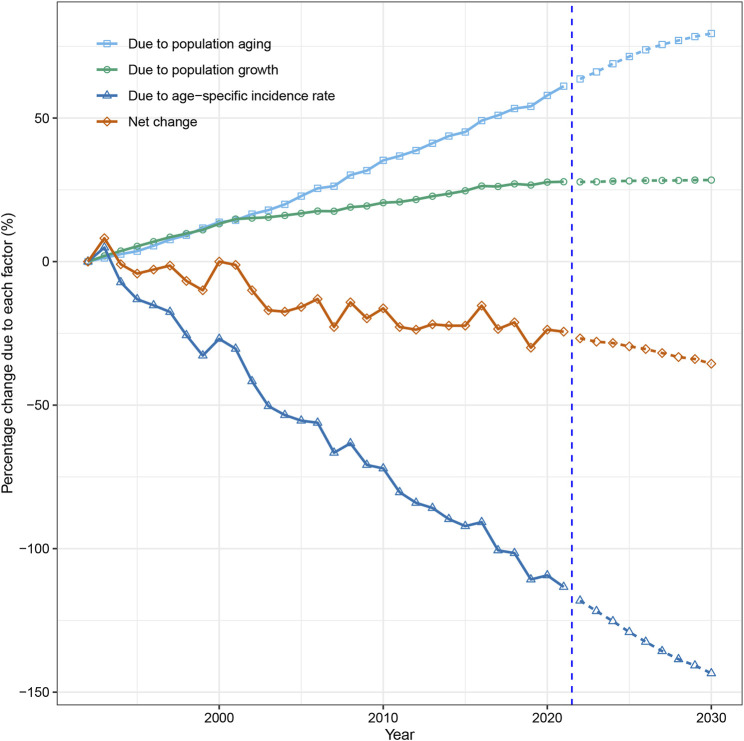
Contribution of changes in population aging, population growth, and age-specific incidence rate to changes in incident cases of esophageal cancer from 1993 to 2030 in Hong Kong men, using 1992 as the reference year. Data in the right of the blue dashed line were the decomposition based on the projected data (Hong Kong, China. 2024).

**FIGURE 6:**
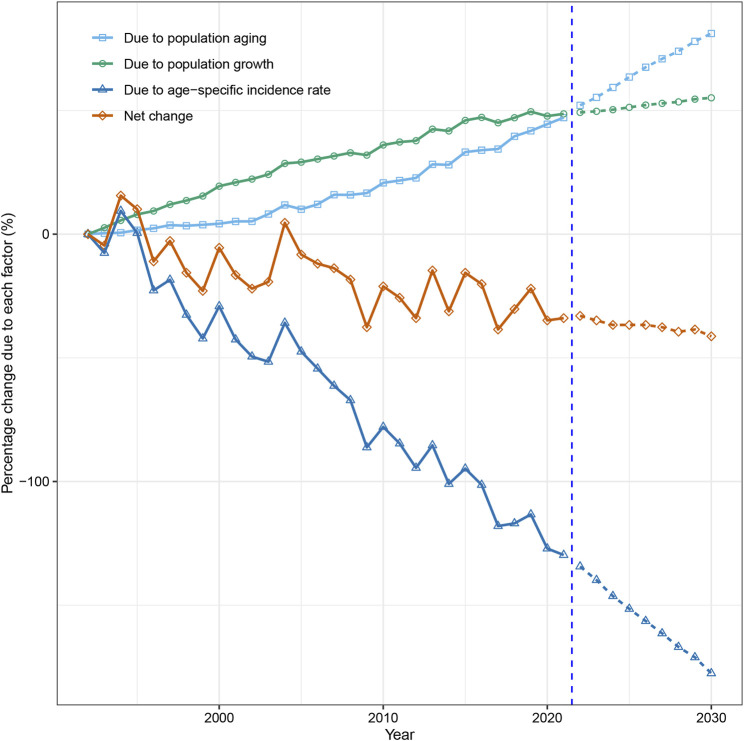
Contribution of changes in population aging, population growth, and age-specific incidence rate to changes in incident cases of esophageal cancer from 1993 to 2030 in Hong Kong women, using 1992 as the reference year. Data in the right of the blue dashed line were the decomposition based on the projected data (Hong Kong, China. 2024).

Our study indicated that while population growth and aging contributed to the rise of esophageal cancer cases, epidemiological factors mitigated this trend and led to a significant decline in esophageal cancer incidence among both sexes in Hong Kong. From 1992 to 2021, the number of new esophageal cancer cases decreased by 24.4% (105 cases) in men and 33.9% (37 cases) in women. Population aging accounted for 61.1% and 47.1% increases in men and women, respectively, while population growth contributed to 27.8% and 48.6% increases in men and women, respectively. However, epidemiological changes significantly decreased esophageal cancer cases, responsible for −113.3% and −129.7% of the difference in men and women, respectively (Figures 5, 6; Supplementary Tables S4, S5).

Our projection and decomposition reveal that epidemiological factors are expected to remain the main driver of the decreasing trend in new esophageal cancer cases in Hong Kong. Our estimates indicate that by 2030, the number of esophageal cancer cases in men and women will decrease by 35.6% (153 cases) and 41.3% (45 cases), respectively, compared to the numbers reported in 1992 (Figures 5, 6; Supplementary Tables S4, S5).

## Discussion

This study provides a comprehensive investigation into the long-term incidence trends of esophageal cancer in Hong Kong over the past three decades. It sheds light on the factors driving these trends. Our findings suggest a significant decline in the incidence of esophageal cancer, primarily due to a reduction in both period and cohort risks of developing the disease. Despite increasing esophageal cancer incidence due to population aging and growth, the progress made in esophageal cancer prevention in Hong Kong has offset this effect. The findings of this study are relevant not only to Hong Kong but also to other regions facing similar epidemiological transitions. Our results further support the effectiveness of public health policies and programs in reducing the incidence of esophageal cancer, particularly those targeting tobacco and alcohol control. Furthermore, our study highlights the importance of continued epidemiological surveillance and research on esophageal cancer in Hong Kong to guide future preventive measures and treatment strategies.

Our study reveals that the decreasing incidence of esophageal cancer in Hong Kong is due to both period and cohort effects. In China, almost half of the incidence of esophageal cancer is attributed to behavioral factors such as smoking, alcohol consumption, and low fruit and vegetable intake [12, 15]. Our findings suggest that the level and trend changes of esophageal cancer incidence in Hong Kong reflect the prevalence of these risk factors. The period effect played a significant role in the decline of esophageal cancer incidence, which can be attributed to implementation of public health policies and programs [19, 28], such as tobacco and alcohol control campaigns, and improved access to medical screening and treatment. Notably, the smoking prevalence rate in Hong Kong has dropped significantly from 23.3% in the early 1980s to 9.5% in 2021 [29], which may be the primary reason for the period effect of esophageal cancer incidence in Hong Kong. In contrast, alcohol consumption has remained stable over the past three decades in Hong Kong, and tax reductions for most types of alcoholic beverages may have led to an increase in alcohol consumption [30]. Thus, alcohol consumption has little positive effect on the period effect of esophageal cancer incidence in Hong Kong.

Cohort effects have also contributed significantly to the declining incidence of esophageal cancer in Hong Kong. Individuals born in the 1950s and 1960s were exposed to higher levels of tobacco and alcohol consumption than their later-born counterparts, which may have played a role in the decreasing incidence of esophageal cancer in subsequent decades [30, 31]. Furthermore, improvements in living standards and medical care could be another explanation for the cohort effect. Those born after World War II had better access to medical care, improved nutrition, and lifestyle factors, which could have contributed to better overall health compared to previous generations. Such living condition improvements may have helped reduce the incidence of esophageal cancer in these cohorts. Therefore, the cohort effect in the decline of esophageal cancer incidence in Hong Kong may be influenced by several factors and is likely multifactorial.

Demographic factors, particularly aging, have played a significant role in the increase in esophageal cancer cases in Hong Kong. The proportion of older individuals in Hong Kong has increased rapidly in the past three decades, with 18.2% of the population aged 65 and over as of 2020, up from 6.5% in 1981 [32]. This demographic trend has contributed to the increase in the incidence of esophageal cancer in the city. Initiatives such as reductions in smoking and drinking rates and changes in healthy lifestyles and eating habits are essential for further reducing the incidence of esophageal cancer in Hong Kong, particularly among older individuals.

In East Asia, including Hong Kong, the primary type of esophageal cancer is SCC [6, 10, 13]. While the smoking rate in Hong Kong is already one of the lowest in the world, further declines in the incidence of esophageal cancer may depend on improvements in other lifestyle factors [7, 30–32]. Reducing alcohol consumption is crucial, as heavy alcohol intake significantly increases the risk of esophageal cancer, particularly SCC, especially when combined with smoking. Additionally, increasing the intake of fruits and vegetables can provide a protective effect against esophageal cancer. Diets rich in fresh produce have been associated with a reduced risk of this disease, likely due to the high levels of vitamins, minerals, and antioxidants that help prevent cellular damage [6]. Conversely, reducing the consumption of pickled foods, which often contain carcinogenic compounds, can further mitigate risk.

Moreover, discouraging the intake of very hot beverages is important, as thermal injury to the esophageal mucosa from such beverages has been linked to an increased risk of esophageal cancer [12, 13, 15, 33]. These dietary and lifestyle changes are essential for continuing the positive trend in reducing esophageal cancer cases in Hong Kong. Public health campaigns and policies that promote healthy eating, moderate alcohol consumption, and awareness of the risks associated with hot beverages can significantly contribute to lowering the incidence of esophageal cancer.

There are several limitations to consider when interpreting the findings of this study. Firstly, although the HKCaR dataset utilized in this study is a reliable source for examining the incidence of esophageal cancer in Hong Kong, the lack of information on esophageal cancer subtypes restricts our ability to explore the incidence trends of different pathological subtypes. Given that SCC is the primary type of esophageal cancer in Hong Kong, this may have minimal impact. Secondly, the absence of patient risk factors exposure information, such as smoking, alcohol consumption, and dietary habits, limits our ability to draw definitive conclusions regarding the reasons for the decline in esophageal cancer incidence in Hong Kong. Collecting such data in future studies would be helpful in better understanding the factors driving the observed trends. Thirdly, while the population structure and size of Hong Kong were derived from UN World Population Prospects, this approach may have introduced biases and underestimated the extent of aging, which could lead to variations in the projections. Finally, although we have identified crucial factors associated with the decline in esophageal cancer incidence, it is possible that other factors, such as changes in diagnostic criteria or advancements in early detection, may have contributed to the observed trends.

### Conclusion

In conclusion, this study highlights a notable decrease in esophageal cancer incidence in Hong Kong, primarily attributed to a decline in period and cohort risks. However, demographic changes such as aging remain a significant risk factor for the disease. Therefore, it is crucial to implement interventions to promote healthy lifestyles and dietary habits, especially among older individuals, to decrease the incidence of esophageal cancer in Hong Kong.

## Data Availability

The esophageal cancer data are available in the Hong Kong Cancer Registry (http://www3.ha.org.hk/cancereg/allages.asp), and the population data for Hong Kong are from the 2022 Revision of World Population Prospects of the United Nations population estimates and projections (https://population.un.org/wpp/).

## Supplementary Material

Supplementary Appendix S1 The decomposition method (Hong Kong, China. 2024).

Supplementary Appendix S2 Projection of future incidence (Hong Kong, China. 2024).

Supplementary Table S1 Wald Chi-square tests for estimable parameters in the Age-Period-Cohort model (Hong Kong, China. 2024).

Supplementary Table S2 Estimated age-specific esophagus cancer cases in Hong Kong men from 1992 to 2030 (Hong Kong, China. 2024).

Supplementary Table S3 Estimated age-specific esophagus cancer cases in Hong Kong women from 1992 to 2030 (Hong Kong, China. 2024).

Supplementary Table S4 Contribution of changes in population aging, population growth, and age-specific incidence rate to the net change of esophagus cancer cases in Hong Kong men from 1992 to 2030 (Hong Kong, China. 2024).

Supplementary Table S5 Contribution of changes in population aging, population growth, and age-specific incidence rate to the net change of esophagus cancer cases in Hong Kong women from 1992 to 2030 (Hong Kong, China. 2024).

## Abbreviations

APC, Age-Period-Cohort; HKCaR, Hong Kong Cancer Registry.
