# Supplementary material for: Evolution of Esophageal Cancer Incidence Patterns in Hong Kong, 1992-2021: An Age-Period-Cohort and Decomposition Analysis
Source: Int J Public Health. 2024 Aug 7;69:1607315. doi: 10.3389/ijph.2024.1607315 (PMC11335483; doi:10.3389/ijph.2024.1607315)
Supplement: Supplementary file 1 [file Table5.pdf]

Table S5. Contribution of changes in population aging, population growth, and age-specific incidence rate to the net change of esophagus cancer cases in Hong Kong women from 1993 to 2030. 1992 was used as the reference year.

Year	Population aging(%)	Population growth(%)	Epidemiological change(%)	Net change(%)
**1993**	0 (0.4)	3 (2.6)	-8 (-7.6)	-5 (-4.6)
**1994**	1 (0.6)	6 (5.6)	10 (9.4)	17 (15.6)
**1995**	2 (1.6)	9 (8.0)	1 (0.5)	11 (10.1)
**1996**	3 (2.4)	10 (9.4)	-25 (-22.7)	-12 (-11.0)
**1997**	4 (3.7)	13 (12.0)	-20 (-18.4)	-3 (-2.8)
**1998**	4 (3.4)	15 (13.6)	-36 (-32.6)	-17 (-15.6)
**1999**	4 (3.8)	17 (15.4)	-46 (-42.2)	-25 (-22.9)
**2000**	5 (4.2)	21 (19.4)	-32 (-29.1)	-6 (-5.5)
**2001**	6 (5.2)	23 (20.9)	-46 (-42.6)	-18 (-16.5)
**2002**	6 (5.2)	24 (22.3)	-54 (-49.5)	-24 (-22.0)
**2003**	9 (8.1)	26 (24.2)	-56 (-51.6)	-21 (-19.3)
**2004**	13 (11.8)	31 (28.6)	-39 (-35.8)	5 (4.6)
**2005**	11 (10.1)	32 (29.1)	-52 (-47.5)	-9 (-8.3)
**2006**	13 (12.1)	33 (30.4)	-59 (-54.4)	-13 (-11.9)
**2007**	17 (15.9)	34 (31.6)	-67 (-61.3)	-15 (-13.8)
**2008**	17 (15.9)	36 (32.9)	-73 (-67.1)	-20 (-18.3)
**2009**	18 (16.6)	35 (32.0)	-94 (-86.2)	-41 (-37.6)
**2010**	23 (20.8)	39 (36.1)	-85 (-77.9)	-23 (-21.1)
**2011**	24 (21.7)	41 (37.3)	-92 (-84.6)	-28 (-25.7)
**2012**	25 (22.7)	41 (37.8)	-103 (-94.5)	-37 (-33.9)
**2013**	31 (28.2)	46 (42.5)	-93 (-85.4)	-16 (-14.7)
**2014**	31 (28.0)	45 (41.7)	-110 (-101)	-34 (-31.2)
**2015**	36 (33.2)	50 (46.0)	-103 (-94.8)	-17 (-15.6)
**2016**	37 (34.0)	51 (47.2)	-110 (-101.4)	-22 (-20.2)
**2017**	38 (34.4)	49 (45.0)	-129 (-118.0)	-42 (-38.5)
**2018**	43 (39.6)	51 (47.1)	-127 (-116.9)	-33 (-30.3)
**2019**	46 (41.8)	54 (49.5)	-124 (-113.3)	-24 (-22.0)
**2020**	48 (44.5)	52 (47.7)	-139 (-127.1)	-38 (-34.9)
**2021**	51 (47.1)	53 (48.6)	-141 (-129.7)	-37 (-33.9)
**2022**	57 (52.1)	54 (49.2)	-146 (-134.4)	-36 (-33.0)
**2023**	60 (55.3)	54 (49.7)	-152 (-139.8)	-38 (-34.9)
**2024**	65 (59.3)	55 (50.3)	-159 (-146.3)	-40 (-36.7)
**2025**	69 (63.5)	56 (51.3)	-165 (-151.5)	-40 (-36.7)
**2026**	74 (67.5)	57 (52.2)	-170 (-156.4)	-40 (-36.7)
**2027**	77 (70.9)	58 (52.9)	-176 (-161.4)	-41 (-37.6)
**2028**	81 (74.0)	58 (53.5)	-182 (-166.9)	-43 (-39.4)

**2029**	85 (78.0)	60 (54.6)	-186 (-171.1)	-42 (-38.5)
**2030**	88 (81.1)	60 (55.1)	-194 (-177.6)	-45 (-41.3)
