# Supplementary material for: Evolution of Esophageal Cancer Incidence Patterns in Hong Kong, 1992-2021: An Age-Period-Cohort and Decomposition Analysis
Source: Int J Public Health. 2024 Aug 7;69:1607315. doi: 10.3389/ijph.2024.1607315 (PMC11335483; doi:10.3389/ijph.2024.1607315)
Supplement: Supplementary file 2 [file Table4.pdf]

Table S4. Contribution of changes in population aging, population growth, and age-specific incidence rate to the net change of esophagus cancer cases in Hong Kong men from 1993 to 2030. 1992 was used as the reference year.

Year	Population aging(%)	Population growth(%)	Epidemiological change(%)	Net change(%)
**1993**	5 (1.3)	8 (2.0)	21 (4.9)	35 (8.1)
**1994**	11 (2.6)	16 (3.7)	-31 (-7.2)	-4 (-0.9)
**1995**	16 (3.6)	23 (5.3)	-56 (-13.1)	-18 (-4.2)
**1996**	24 (5.5)	30 (6.9)	-65 (-15.2)	-12 (-2.8)
**1997**	33 (7.7)	37 (8.5)	-75 (-17.5)	-6 (-1.4)
**1998**	39 (9.2)	42 (9.7)	-110 (-25.7)	-29 (-6.7)
**1999**	50 (11.7)	48 (11.1)	-141 (-32.7)	-43 (-10.0)
**2000**	59 (13.7)	57 (13.2)	-116 (-26.9)	0 (0.0)
**2001**	62 (14.4)	64 (14.8)	-131 (-30.4)	-5 (-1.2)
**2002**	71 (16.5)	65 (15.2)	-179 (-41.7)	-43 (-10.0)
**2003**	77 (17.9)	66 (15.4)	-216 (-50.3)	-73 (-17.0)
**2004**	86 (19.9)	69 (16.1)	-230 (-53.5)	-75 (-17.4)
**2005**	98 (22.8)	72 (16.8)	-238 (-55.4)	-68 (-15.8)
**2006**	110 (25.5)	76 (17.6)	-241 (-56.1)	-56 (-13.0)
**2007**	113 (26.3)	75 (17.5)	-286 (-66.6)	-98 (-22.8)
**2008**	130 (30.1)	82 (19.0)	-272 (-63.3)	-61 (-14.2)
**2009**	136 (31.7)	83 (19.4)	-305 (-70.8)	-85 (-19.8)
**2010**	152 (35.3)	88 (20.5)	-310 (-72.1)	-70 (-16.3)
**2011**	158 (36.8)	89 (20.8)	-346 (-80.4)	-98 (-22.8)
**2012**	166 (38.7)	93 (21.6)	-361 (-84.0)	-102 (-23.7)
**2013**	177 (41.2)	98 (22.8)	-369 (-85.8)	-94 (-21.9)
**2014**	188 (43.7)	102 (23.6)	-386 (-89.7)	-96 (-22.3)
**2015**	194 (45.2)	106 (24.7)	-396 (-92.1)	-96 (-22.3)
**2016**	211 (49.1)	113 (26.3)	-390 (-90.8)	-66 (-15.3)
**2017**	219 (50.9)	112 (26.2)	-433 (-100.6)	-101 (-23.5)
**2018**	229 (53.3)	116 (27.1)	-437 (-101.5)	-91 (-21.2)
**2019**	233 (54.1)	115 (26.7)	-476 (-110.7)	-129 (-30.0)
**2020**	249 (57.9)	119 (27.7)	-470 (-109.3)	-102 (-23.7)
**2021**	263 (61.1)	120 (27.8)	-487 (-113.3)	-105 (-24.4)
**2022**	274 (63.6)	119 (27.8)	-508 (-118.1)	-115 (-26.7)
**2023**	284 (66.1)	119 (27.8)	-524 (-121.8)	-120 (-27.9)
**2024**	296 (68.9)	120 (28.0)	-539 (-125.3)	-122 (-28.4)
**2025**	307 (71.4)	121 (28.1)	-555 (-129.1)	-127 (-29.5)
**2026**	317 (73.8)	121 (28.2)	-570 (-132.5)	-131 (-30.5)
**2027**	325 (75.6)	121 (28.2)	-584 (-135.7)	-137 (-31.9)
**2028**	331 (77.0)	122 (28.3)	-596 (-138.5)	-143 (-33.3)

**2029**	337 (78.4)	122 (28.4)	-605 (-140.7)	-146 (-34.0)
**2030**	342 (79.5)	122 (28.4)	-617 (-143.4)	-153 (-35.6)
