# Supplementary material for: Evolution of Esophageal Cancer Incidence Patterns in Hong Kong, 1992-2021: An Age-Period-Cohort and Decomposition Analysis
Source: Int J Public Health. 2024 Aug 7;69:1607315. doi: 10.3389/ijph.2024.1607315 (PMC11335483; doi:10.3389/ijph.2024.1607315)
Supplement: Supplementary file 3 [file Table1.pdf]

Table S1. Wald Chi-square tests for estimable parameters in the APC model

Null Hypothesis	Males		Females	
	Chi-square	P-value	Chi-square	P-value
**NetDrift = 0**	86.64	<0.001	73.75	<0.001
**All Age Deviations = 0**	1052.47	<0.001	76.45	<0.001
**All Period RR = 1**	99.80	<0.001	78.81	<0.001
**All Cohort RR = 1**	1541.64	<0.001	496.19	<0.001
**All Local Drifts = Net Drift**	51.00	<0.001	38.96	<0.001
