# Supplementary material for: Evolution of Esophageal Cancer Incidence Patterns in Hong Kong, 1992-2021: An Age-Period-Cohort and Decomposition Analysis
Source: Int J Public Health. 2024 Aug 7;69:1607315. doi: 10.3389/ijph.2024.1607315 (PMC11335483; doi:10.3389/ijph.2024.1607315)
Supplement: Supplementary file 4 [file Presentation1.pdf]

Appendix S1. The decomposition method

The population decomposition algorithm has been described in detail in the papers by Cheng et al. [1,2].

Briefly, take the difference in newly diagnosed esophagus cancer cases between 1992 and 2021 in Hong Kong. We can decompose the net change of incident cases into the contribution of population growth, population aging, and age-specific incidence rate.

The age groups were divided using 5-year increments from 20-24 years to 85 plus (we included older people aged ⩾85 years as the 85-89 years age group, which was recorded as only one group in the database). Let d_{ij}, n_{ij}, m_{ij} and s_{ij} denote the incident cases, population size, age-specific rate of incidence, and population proportion in the ith age group of the year j, respectively, (i = 1, 2, …,12; j = 1, 2). Let D_1 and D_2, N_1 and N_2, P_1 and P_2 represent the total incident cases, population size, and crude rate of incidence in 1992 and 2021.

Using M_p, M_a, and M_m to represent the main effects of the changes in population size, age structure, and incidence rate, and I_{pa}, I_{pm}, I_{am}, and I_{pam} to represent their two-way and three-way interactions, respectively. In the case of 1992 as the reference year, these terms are calculated as follows:

$$M_p = \sum_{i=1}^{12}(N_2 - N_1)s_{i1}m_{i1}$$

$$M_a = \sum_{i=1}^{12} N_1(s_{i2} - s_{i1})m_{i1}$$

$$M_m = \sum_{i=1}^{12} N_1 s_{i1}(m_{i2} - m_{i1})$$

$$I_{pa} = \sum_{i=1}^{12}(N_2 - N_1)(s_{i2} - s_{i1})m_{i1}$$

$$I_{pm} = \sum_{i=1}^{12}(N_2 - N_1)s_{i1}(m_{i2} - m_{i1})$$

$$I_{am} = \sum_{i=1}^{12} N_1(s_{i2} - s_{i1})(m_{i2} - m_{i1})$$

$$I_{pam} = \sum_{i=1}^{12}(N_2 - N_1)(s_{i2} - s_{i1})(m_{i2} - m_{i1})$$

Here, a simplification needs to be made, assuming that the interactions are equally distributed, then the contribution of the three factors can be calculated as follows:

$$A = M_a + ½I_{am} + ½I_{pa} + ⅓I_{pam}$$

$$P = M_p + \frac{1}{2} I_{pm} + \frac{1}{2} I_{pa} + \frac{1}{3} I_{pam}$$

$$M = M_m + \frac{1}{2} I_{pm} + \frac{1}{2} I_{am} + \frac{1}{3} I_{pam}$$

Here, A represents the contribution of population aging, P represents the contribution of population growth, M represents the contribution of the age-specific death rate, and net change represents total change. The contribution of each factor divided by D_I and multiplied by 100 is the percentage of the respective contribution.

References

1. Cheng XJ, Yang Y, Schwebel DC, Liu ZY, Li L, Cheng PX, et al. Population Ageing and Mortality during 1990-2017: A Global Decomposition Analysis. PLoS Med (2020) 17(6):e1003138. doi:10.1371/journal.pmed.1003138

2. Cheng XJ, Tan LH, Gao YY, Yang Y, Schwebel DC, Hu GQ. A New Method to Attribute Differences in Total Deaths between Groups to Population Size, Age Structure and Age-Specific Mortality Rate. PLoS One (2019) 14(5):e0216613. doi:10.1371/journal.pone.0216613
