# Supplementary material for: Evolution of Esophageal Cancer Incidence Patterns in Hong Kong, 1992-2021: An Age-Period-Cohort and Decomposition Analysis
Source: Int J Public Health. 2024 Aug 7;69:1607315. doi: 10.3389/ijph.2024.1607315 (PMC11335483; doi:10.3389/ijph.2024.1607315)
Supplement: Supplementary file 5 [file Table2.pdf]

Table S2. Estimated age-specific esophagus cancer cases in Hong Kong men from 1992 to 2030

Year	Number of age-specific esophagus cancer cases													
	20-24	25-29	30-34	35-39	40-44	45-49	50-54	55-59	60-64	65-69	70-74	75-79	80-84	85+	Total
**1992**	3	2	2	4	7	15	32	59	90	78	63	53	13	9	430
**1993**	1	0	0	6	14	17	36	59	97	80	71	54	24	6	465
**1994**	0	0	0	5	13	26	34	62	90	75	55	38	23	5	426
**1995**	0	1	1	5	3	17	40	60	72	92	51	40	19	11	412
**1996**	0	1	0	5	9	18	22	51	86	83	66	44	21	12	418
**1997**	0	0	0	4	17	15	32	47	69	98	75	27	30	10	424
**1998**	0	0	1	1	10	23	25	44	71	84	57	52	21	12	401
**1999**	0	0	0	2	10	23	28	35	57	71	80	48	17	16	387
**2000**	0	0	0	3	13	20	51	48	49	82	77	45	28	14	430
**2001**	1	0	0	2	7	26	40	57	67	71	72	47	18	17	425
**2002**	0	0	0	0	10	14	43	36	61	57	76	43	34	13	387
**2003**	0	0	0	1	7	24	40	35	53	56	68	41	24	8	357
**2004**	0	0	0	2	7	11	31	50	42	55	64	50	28	15	355
**2005**	0	0	0	3	3	16	39	54	39	50	66	45	27	20	362
**2006**	0	0	0	0	9	15	36	56	40	53	49	58	35	23	374
**2007**	0	0	0	2	5	23	28	47	44	46	48	44	23	22	332
**2008**	1	0	0	1	3	18	34	55	49	47	59	55	30	17	369
**2009**	0	0	0	1	1	15	25	46	51	51	65	48	26	16	345
**2010**	0	0	3	0	3	12	40	46	49	45	57	50	33	22	360
**2011**	0	0	0	0	6	15	24	50	54	43	32	52	31	25	332
**2012**	0	0	2	1	2	9	25	35	57	49	45	44	29	30	328

**2013**	0	0	0	1	5	10	23	51	56	50	41	43	28	28	336
**2014**	0	0	0	1	3	7	29	36	53	57	42	42	41	23	334
**2015**	1	0	0	1	7	11	16	50	54	57	50	39	28	20	334
**2016**	0	1	0	0	3	11	22	47	72	77	45	31	39	16	364
**2017**	0	0	0	0	2	8	21	46	54	51	47	38	28	34	329
**2018**	0	0	0	2	3	8	20	34	58	61	55	40	30	28	339
**2019**	0	0	1	0	6	8	14	32	54	53	47	32	31	23	301
**2020**	0	0	0	1	4	9	17	34	65	64	52	39	26	17	328
**2021**	0	0	0	2	3	9	14	35	47	59	63	37	29	27	325
**2022**	0	0	0	1	3	7	14	30	52	57	58	37	28	28	315
**2023**	0	0	0	1	3	7	14	28	49	57	57	40	26	28	310
**2024**	0	0	0	1	3	6	14	26	47	57	56	43	26	29	308
**2025**	0	0	0	1	3	6	13	24	44	57	55	46	25	29	303
**2026**	0	0	0	1	3	6	13	23	41	55	54	49	25	29	299
**2027**	0	0	0	1	2	6	13	22	37	53	54	50	27	28	293
**2028**	0	0	0	1	2	6	12	21	34	50	55	49	29	28	287
**2029**	0	0	0	1	2	6	12	21	32	48	55	48	31	28	284
**2030**	0	0	0	1	2	6	11	20	29	45	54	47	34	28	277
