# Supplementary material for: Evolution of Esophageal Cancer Incidence Patterns in Hong Kong, 1992-2021: An Age-Period-Cohort and Decomposition Analysis
Source: Int J Public Health. 2024 Aug 7;69:1607315. doi: 10.3389/ijph.2024.1607315 (PMC11335483; doi:10.3389/ijph.2024.1607315)
Supplement: Supplementary file 6 [file Table3.pdf]

Table S3. Estimated age-specific esophagus cancer incident cases in Hong Kong women from 1992 to 2030

Year	Number of age-specific esophagus cancer cases														
	20-24	25-29	30-34	35-39	40-44	45-49	50-54	55-59	60-64	65-69	70-74	75-79	80-84	85+	Total
**1992**	1	0	0	1	1	2	5	5	15	24	10	18	12	15	109
**1993**	0	2	0	1	0	3	7	6	8	19	20	14	15	9	104
**1994**	0	2	0	1	2	4	5	7	12	20	24	25	13	11	126
**1995**	1	0	1	1	3	5	5	4	10	24	26	20	10	10	120
**1996**	0	0	0	1	4	6	2	8	8	9	12	17	16	14	97
**1997**	0	1	0	1	3	7	1	9	10	17	13	14	12	18	106
**1998**	0	1	1	1	0	3	6	7	3	13	20	15	13	9	92
**1999**	0	0	1	0	0	3	4	5	6	6	16	17	16	10	84
**2000**	0	0	0	0	1	2	6	6	7	19	15	16	17	14	103
**2001**	0	0	0	0	2	3	4	4	5	12	9	17	17	18	91
**2002**	1	0	0	0	1	1	0	6	5	15	12	16	15	13	85
**2003**	0	0	0	0	1	2	6	5	6	11	16	15	12	14	88
**2004**	0	0	0	0	1	5	2	6	4	15	18	30	18	15	114
**2005**	0	0	1	0	0	2	8	8	12	12	13	10	11	23	100
**2006**	0	0	0	0	2	1	5	7	8	15	14	18	10	16	96
**2007**	0	0	0	1	1	1	6	8	6	5	15	17	18	16	94
**2008**	0	0	0	0	0	1	3	8	10	9	8	13	17	20	89
**2009**	0	0	0	1	0	0	2	3	6	5	13	10	10	18	68
**2010**	0	0	0	0	1	1	3	4	9	5	15	11	10	27	86
**2011**	0	0	0	0	0	3	1	6	11	6	12	14	9	19	81
**2012**	1	0	1	0	0	4	2	4	6	7	8	13	13	13	72

**2013**	0	1	0	0	0	3	4	6	8	9	8	19	13	22	93
**2014**	0	0	1	0	2	2	1	5	5	7	10	12	15	15	75
**2015**	0	0	0	0	0	3	5	4	15	9	10	10	18	18	92
**2016**	0	0	1	1	2	1	3	6	8	6	10	20	10	19	87
**2017**	0	1	0	0	1	1	2	4	7	8	10	8	10	15	67
**2018**	0	1	0	0	0	2	4	5	6	11	5	7	15	20	76
**2019**	0	0	0	2	0	0	1	2	7	20	13	13	12	15	85
**2020**	0	0	1	0	0	3	2	8	6	5	8	10	11	17	71
**2021**	0	0	1	1	1	3	2	3	5	9	7	10	14	16	72
**2022**	0	0	0	0	1	1	2	4	6	9	11	10	10	19	73
**2023**	0	0	0	0	1	1	2	3	6	8	11	11	10	18	71
**2024**	0	0	0	0	1	1	2	3	5	8	11	11	9	18	69
**2025**	0	0	0	0	1	1	2	3	5	8	10	12	9	18	69
**2026**	0	0	0	0	1	1	2	3	5	8	10	12	9	18	69
**2027**	0	0	0	0	1	1	2	3	5	7	10	12	9	18	68
**2028**	0	0	0	0	1	1	2	3	4	7	9	12	10	17	66
**2029**	0	0	0	0	1	1	2	3	4	7	9	12	11	17	67
**2030**	0	0	0	0	1	1	2	3	4	6	9	11	11	16	64
