# Supplementary material for: Evolution of Esophageal Cancer Incidence Patterns in Hong Kong, 1992-2021: An Age-Period-Cohort and Decomposition Analysis
Source: Int J Public Health. 2024 Aug 7;69:1607315. doi: 10.3389/ijph.2024.1607315 (PMC11335483; doi:10.3389/ijph.2024.1607315)
Supplement: Supplementary file 7 [file Presentation2.pdf]

Appendix S2. Projection of future incidence

The future incident cases of esophagus cancer in Hong Kong were projected using the Bayesian age-period-cohort analysis with integrated nested Laplace approximations (INLA). Compared to the classical age-period-cohort model, the Bayesian approach attributes separate effects to age, period, and cohort, and extrapolates these effects to make projections. The Bayesian approach leads to more precise estimates of incidence rates than the corresponding maximum likelihood estimates used in the classical model. In addition, since the Bayesian approach does not depend on strong parametric assumptions like the classical approach, it is the only current method to achieve nonarbitrary and sensible projections [1].

Based on the expectation that effects adjacent in time might be similar, the Bayesian inference in age-period-cohort model applies the second-order random walk (RW2) for smoothing priors of age, period, and cohort effects and to project posterior incidence rates. According to this model, each point of effect is predicted by linear extrapolation from its two immediate predecessors plus a random variance from a normal distribution with a mean zero. The INLA is used with this Bayesian age-period-cohort model to approximate the marginal posterior distributions avoiding any mixing and convergence issues introduced by Markov chain Monte Carlo (MCMC) sampling techniques traditionally used in the Bayesian approach. The Bayesian age-period-cohort analysis was conducted by an R-package BAPC available from R-forge (http://r-forge.r-project.org/) [2].

We used inverse gamma priors with shape parameter a = 1 and rate parameter b = 0.00005 for the RW2 variance parameters of the time effects (age, period, and cohort) and an inverse gamma prior with a = 1 and b = 0. 005 for the overdispersion variance. It has been found that the results of age-specific and age-standardized predictions have little prior sensitivity.

We prepared age-specific incident cases of esophagus cancer (from 1992 to 2020)

and Hong Kong population data (from 1992 to 2030), followed by a 9-year (from 2022 to 2030) retrospective projection using the BAPC function in the R package BAPC.

References

1. Jacobs D, Huang H, Olino K, Weiss S, Kluger H, Judson BL, et al. Assessment of Age, Period, and Birth Cohort Effects and Trends in Merkel Cell Carcinoma Incidence in the United States. JAMA Dermatol (2021) 157(1):59-65. doi:10.1001/jamadermatol.2020.4102
2. Riebler A, Held L. Projecting the Future Burden of Cancer: Bayesian Age-Period-Cohort Analysis with Integrated Nested Laplace Approximations. Biom J (2017) 59(3):531-49. doi:10.1002/bimj.201500263
